# Discovery of Novel Peptides as DPP-IV Inhibitors: *In Silico* Screening, Molecular Dynamics Simulations, and Preliminary *In Vitro* Evaluation

**DOI:** 10.1021/acsomega.6c08022

**Published:** 2026-09-18

**Authors:** Burak Calis, Fernando Berton Zanchi, Beyza Canakcimaksutoglu, Ahmet E. Yetiman, Ozkan Fidan

**Affiliations:** † Department of Bioengineering, Graduate School, Abdullah Gul University, Kayseri, Türkiye 38080; ‡ Bioinformatic and Medicinal Chemistry Laboratory, Fiocruz/RO, Porto Velho, Rondonia Brazil 76812-245; § Department of Food Engineering, Faculty of Engineering, 52958 Erciyes University, Kayseri, Türkiye 38030; ∥ Department of Bioengineering, Faculty of Life and Natural Sciences, Abdullah Gul University, Kayseri, Türkiye 38080

## Abstract

Type 2 diabetes mellitus (T2DM) is the most common metabolic disorder characterized by impaired insulin secretion, which leads to obesity. The incretin hormone glucagon-like peptide-1 (GLP-1) has a significant role in glucose homeostasis, but its native form in the human body is highly susceptible to proteolytic degradation by dipeptidyl peptidase IV (DPP-IV). In this study, a peptide library was constructed from antimicrobial peptide databases to discover novel DPP-IV inhibitors based on structural similarity to that of native GLP-1. A total of 26 candidate peptides were shortlisted and screened using *in silico* analysis, including molecular docking, pharmacokinetic profiling, and molecular dynamics simulations. Particularly, two of the candidate antimicrobial peptides showed potent binding affinity to DPP-IV, with free energy scores of −15.6 and −16.5 kcal/mol, surpassing the binding affinity of native GLP-1 for DPP-IV. These peptides also exhibited lower predicted binding affinities for the GLP-1 receptor (GLP-1R) than for DPP-IV, suggesting a potential selective inhibitory effect and, consequently, a lower predicted likelihood of interfering with native GLP-1 signaling. Pharmacokinetic characteristics of the peptides were promising with an optimal absorption, distribution, metabolism, excretion, and toxicity (ADMET) profile, along with prolonged half-life and stability. Because of its better membrane permeability and more favorable predicted DPP-IV/GLP-1R binding preference, peptide 3 was further evaluated for downstream experimental validation. The biological activity test showed that peptide 3 successfully exhibited a 36.2% inhibition rate against DPP-IV *in vitro*. These findings indicate that both peptides 3 and 10 are promising DPP-IV inhibitor candidates.

## Graphical abstract

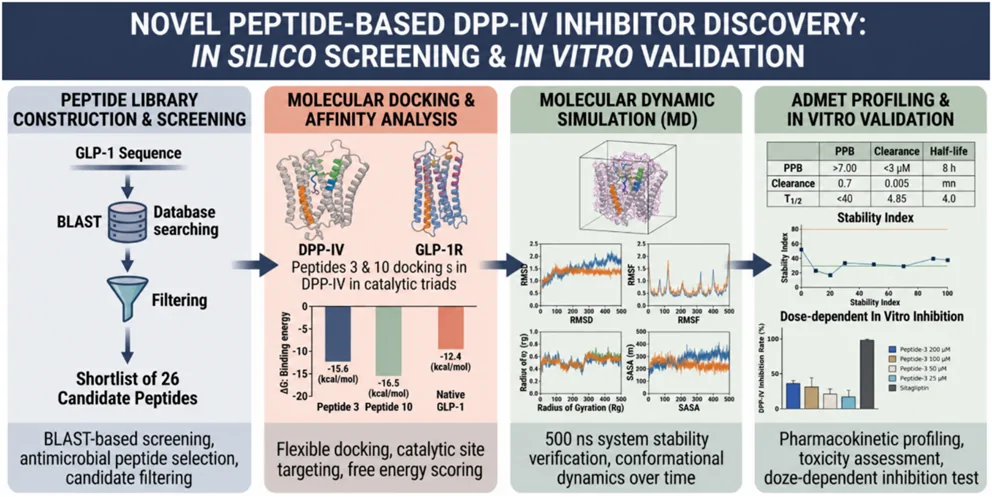

## 1 Introduction

Diabetes and obesity are two interrelated health problems that have become increasingly common around the world. Diabetes is a chronic condition that affects the body’s capacity to produce or use insulin, which is a hormone that regulates blood sugar. There are two main types of diabetes: type 1 diabetes mellitus (T1DM), an autoimmune disorder that typically develops in childhood, and type 2 diabetes mellitus (T2DM), which is often due to lifestyle factors, such as poor diet, lack of physical activity, and obesity.^1^ The incidence of diabetes in developed countries was reported as approximately 5–10% of the population. 90–95% of diabetes cases worldwide are T2DM.^2^

Currently, the primary treatment method against T2DM is insulin therapy. In addition, the growing development of biotechnological drugs has become a promising research area in treatment. One of the biotechnological drugs is the recombinant production of glucagon-like peptide-1 (GLP-1). GLP-1 is an endogenous incretin and plays an important role in glucose homeostasis; it is a 30-amino-acid-containing peptide secreted from intestinal L-cells depending on food intake. It is produced by post-translational processing of preproglucagon. It binds and activates the GLP-1 receptor (GLP-1R), which belongs to the class B family of G-protein-coupled receptors (GPCRs).^3^ While GLP-1R is found in many organs, including the pancreas, brain, heart, kidney, and gastrointestinal tract, it is known to be expressed only in pancreatic β-cells and rarely in α- and δ-cells.^4^ Although GLP-1R in α-cells is less than 0.2% of that in β-cells, GLP-1 inhibits glucagon secretion by 50% through the modulation of calcium channel activity.^5^ Ultimately, GLP-1 exerts its effects on glucose metabolism by increasing insulin secretion in a glucose-dependent manner.^6^

The native GLP-1 peptide in the human body has a very short half-life of 1–2 min due to rapid metabolic degradation by proteases, such as neutral endopeptidase 24.11 (NEP 24.11) and predominantly dipeptidyl peptidase IV (DPP-IV). DPP-IV cleaves the peptide bond at Ala8-Glu9, and the resulting metabolite GLP-1­(9-36)-NH_2_ shows a 100-fold lower binding affinity than the intact peptide. In addition, this resulting metabolite has negligibly low agonistic activity.^7^ Thus, GLP-1 analogues and DPP-IV inhibitors have been developed to overcome GLP-1 degradation. Exenatide, lixisenatide, semaglutide, tirzepatide, and dulaglutide are some of the FDA-approved peptide-based GLP-1 analogues with prolonged half-life and enhanced binding affinity to GLP-1R.^8^

Bioactive peptides and antimicrobial peptides have been studied as potential DPP-IV inhibitors. For instance, blood glucose level reducing effects in T2DM mouse models demonstrated the biological efficacy of DPP-IV inhibitor peptides.^9^ Among previous supportive studies, novel peptides from *M. integrifolia* have been identified as promising antidiabetic compounds using *in silico* analysis. Particularly, *M. integrifolia* antimicrobial protein 2 (MiAMP2) was investigated, and an *in silico* digestion of this protein yielded the tetrapeptide AESE, which was subsequently reported to exhibit DPP-IV inhibitory activity.^10^

This study mainly focused on screening of relatively longer natural peptides based on their similarity with native GLP-1 and evaluating them for their DPP-IV inhibition potency through *in silico* tools. A total of 26 peptides were shortlisted for the ultimate purpose of defining the best candidates based on structural similarity with native GLP-1 and were subsequently screened against DPP-IV using molecular docking analysis. Two hit peptides were selected as the most promising ones with their stronger binding affinity for DPP-IV. Pharmacokinetic evaluations showed their potential to be used as a drug molecule. Additionally, molecular dynamics simulations were performed to confirm binding affinity, stability, and structure compactness.

## 2 Methodology

### 2.1 Peptide Library Construction

The peptide sequence “HAEGTFTSDVSSYLEGQAAKEFIAWLVKGR” was divided into two fragments of 17 and 13 amino acids (HAEGTFTSDVSSYLEGQ and AAKEFIAWLVKGR). These sequences, along with GLP-1, were analyzed using BLASTp against the Swiss-Prot and NCBI databases in Python 3.9. For the full-length sequences, 73 similar matches were retrieved from the Swiss-Prot database (Table S1), and 50 were obtained from NCBI (Table S2); while some displayed 100% identity, others exhibited similarities below 70%. For the 17-amino-acid fragment, Swiss-Prot yielded 11 similar protein sequences (Table S3), only one of which had 82% similarity, while the remaining sequences showed 100% identity and were subsequently excluded. No matches were found in the NCBI database for the same fragment. For the 13-amino-acid fragment, neither database yielded any suitable matches. The expected value threshold was strictly set to 0.004 to eliminate random similarities and filter out false-positive alignments for these short amino acid sequences. FASTA-formatted antimicrobial peptide sequences were retrieved from APD3 (March 20, 2023, version). Additionally, reviewed toxin proteins from UniProtKB were downloaded on May 3, 2023. Local BLAST databases were established for APD, PeptideAtlas, and UniProtKB. All BLAST analyses were evaluated on the basis of identity, query coverage, and subject similarity.

Protein sequences from honeybee, *B. burgdorferi*, *Streptococcus*, fission yeast, cow, *Candida albicans*, *C. elegans*, *Gallus gallus*, *Drosophila*, zebrafish, *Halobacterium*, *Leptospira interrogans*, mouse, *M. tuberculosis*, *P. falciparum*, pig, rat, *S. aureus* COL, and *S. aureus* HG001 were obtained from the PeptideAtlas database (https://peptideatlas.org/). The peptides were compiled into an Excel spreadsheet, resulting in a total of 70 GLP-1 variants (Table S4), which included 13 truncated variants of 13 amino acids (Table S5) and 57 truncated variants of 17 amino acids (Table S6). Humanlike sequences were excluded, while venom-derived peptides were prioritized. Native GLP-1-like peptides undergo rapid enzymatic cleavage by DPP-IV, thus acting as transient substrates rather than as stable inhibitors.^11^ In contrast, venom-derived peptides were prioritized because they provide structurally rigid and disulfide-rich scaffolds that can offer high target affinity, along with enhanced resistance to proteolytic degradation.^12^ Subsequently, peptides exhibiting higher sequence identity were selected. In addition, 19 GLP-1 variants were obtained from UniProt and APD. Furthermore five truncated peptides each for the 13- and 17-amino-acid lengths were selected from APD, PeptideAtlas, and Swiss-Prot. After eliminating redundant sequences, a final data set comprising 26 peptides from 18 species was shortlisted. Signal and propeptide sequences were excluded from specific peptides to enhance specificity in subsequent analyses as they do not contribute to peptide interactions and are removed via post-translational modifications. The source species are listed in Table 1.

**Table 1:** Binding Free Energy Scores of the Candidate Peptides toward DPP-IV and GLP-1R

peptide ID	source	amino acid sequence	binding free energy with DPP-IV (kcal/mol)	binding free energy with GLP-1R (kcal/mol)
Peptide 1	*Apis mellifera* (Honeybee)	EFLAWLYNESSVK	–12.1	–8.2
Peptide 2	*Anguilla anguilla* (European eel)	HAEGTYTSDVSSYLQDQ	–14.8	–7.5
Peptide 3	*Apis mellifera* (Honeybee)	EIKPSVLIGASAAAGAFTTDVLK	**–15.6**	–9.7
Peptide 4	*Pagrus major* (Red seabream)	FFGWLIKGAIHAGKAIHGLIHRRRH	–12.2	–7.9
Peptide 5	*Gallus gallus* (Chicken)	TFVASEFIDWLIQEGEATTR	–10.1	–8.3
Peptide 6	*Apis mellifera* (Honeybee)	FDVNITELPDEIDLASYIEGR	–12.8	–9.9
Peptide 7	*Chrysophrys major* (Goldband snapper)	FFGWLIRGAIHAGKAIHGLIHRRRH	–13	–7.9
Peptide 8	*Bothrops jararaca* (Jararaca snake)	DCPSDWSPYEGHCYKHFIKWMNNEDAERFC	–13.2	–10.9
Peptide 9	*Halobacterium*	EEFDEWAASGR	–9.9	–8.3
Peptide 10	*Urodacus yaschenkoi* (Australian scorpion)	GIWSWIKKTAKKVWNSDVAKKLKGKALNVAKDFVAEKIGATPA	**–16.5**	–11.4
Peptide 11	*Clarias gariepinus* (African catfish)	HSDGIFTDSYSRYRKQMAVKKYLAAVLGRRYRQRFRNK	–14.7	–12.3
Peptide 12	*Heterometrus spinifer* (Asian forest scorpion)	GVWDWLKKTAKNVWNSDIVKQLKGKAINAAKNYVAEKIGATPS	–13.1	–13.8
Peptide 13	*Heterometrus spinifer*	GVWDWLKKTAKNVWNSDIVKQLKGKAINAAKNYVAEKIGATPS	–13.4	–12
Peptide 14	*Psalmopoeus cambridgei* (Trinidad chevron tarantula)	GACRWFLGGCKSTSDCCEHLSCKMGLDYCAWDGTF	–11.5	–6.6
Peptide 15	*Heteroscodra maculata* (Togo starburst tarantula)	ECRYLFGGCSSTSDCCKHLSCRSDWKYCAWDGTFS	–13.5	–11.6
Peptide 16	*Conus pennaceus* (Pennant cone-sea snail)	GCCSRPPCALSNPDYC	–11.5	–11.7
Peptide 17	*Conus pennaceus*	GCCSLPPCALSNPDYC	–13.1	–8
Peptide 18	*Conus episcopatus* (Bishop’s cone)	GCCSDPRCNMNNPDYC	–10.8	–7.3
Peptide 19	*Conus aulicus* (Prince cone)	GCCSYPPCFATNSDYC	–9.9	–9.8
Peptide 20	*Conus aulicus*	GCCSYPPCFATNPDC	–13.4	–11
Peptide 21	*Conus victoriae* (Queen’s cone)	GCCSNPACMVNNPQIC	–9.9	–8.8
Peptide 22	*Conus marmoreus* (Marble cone)	GCCSNPPCYANNQAYCN	–14	–9.3
Peptide 23	*Conus marmoreus*	GCCSHPACSVNNPDIC	–12.4	–11.4
Peptide 24	*Conus victoriae*	GCCSDPPCIANNPDLC	–13.7	–8.9
Peptide 25	*Conus pennaceus*	GCCSHPPCFLNNPDYC	–11.3	–6.6
Peptide 26	*Conus textile* (Textile cone)	GCCSRPPCIANNPDLC	–12.3	–7.4
Native GLP-1	Human	HAEGTFTSDVSSYLEGQAAKEFIAWLVKGR	–12.4	–11.2
Heterin-1 derivative	Peptide-based GLP-1 analogue	HGEGVWNSDIVKQLEGKAINAAKEFVAEKIGATPS	–13.9	–8.7
Dulaglutide	FDA-approved GLP-1 analogue	HGEGTFTSDVSSYLEEQAAKEFIAWLVKGG	–14.7	–11
Exenatide	FDA-approved GLP-1 analogue	HGEGTFTSDLSKQMEEEAVRLFIEWLKNGGPSSGAPPPS	–14.6	–14.6
Lixisenatide	FDA-approved GLP-1 analogue	HGEGTFTSDLSKQMEEEAVRLFIEWLKNGGPSSGAPPSKKKKKK	–16.9	–10.4
Semaglutide	FDA-approved GLP-1 analogue	HAEGTFTSDVSSYLEGQAAKEFIAWLVRGRG	–13.6	–9.6

### 2.2 Molecular Docking Studies

Among the shortlisted peptides, the ones obtained from the PeptideAtlas database were subjected to 3D modeling using I-TASSER (https://seq2fun.dcmb.med.umich.edu/I-TASSER/) online software. According to the C-score primarily, TM-score, and RMSD value, the highest-ranked one was chosen among the predicted 3D models. DPP-IV (PDB Accession number: 3D4L) and GLP-1R (PDB Accession number: 7S15) 3D protein structures were retrieved from the Protein Data Bank (https://www.rcsb.org/). All of the eligible peptides, DPP-IV, and GLP-1R structures were collected as PDB files, and the peptides were subjected to molecular docking with both DPP-IV and GLP-1R. An online tool, HADDOCK 2.4 (https://wenmr.science.uu.nl/), was used to analyze docking interactions. Active residues of DPP-IV and GLP-1R were identified based on their interaction with native GLP-1 in the human body. According to previous findings, specifically Ser630, Asp708, His740, Tyr547, Glu205, and Glu206 residues of DPP-IV were selected as active residues in the docking protocol parameters because of their critical roles in proteolytic activity and ligand recognition. Ser630, Asp708, and His740 were described as the catalytic triad and facilitate substrate cleavage.^13^ Their position provides a cave-like projection surrounded by hydrophobic residues. Thus, the S1 pocket of DPP-IV, comprising Ser630, Asp708, and His740, was characterized by a hydrophobic environment and influences binding affinity and specificity.^14^ Tyr547, positioned in the hydrolase domain, has a role in substrate stabilization and β-propeller domain members, highly conserved DPP-IV residues Glu205 and Glu206, are crucial for peptide-substrate alignment with an optimal cleavage orientation.^13^ For the peptide ligands, the whole amino acid sequences were described as active residues during the docking protocol. The potency of interaction was scored based on exhibited strong hydrogen bond, electrostatic and hydrophobic interactions between the key amino acids of DPP-IV, and candidate peptide ligands.

Since eligible peptides were selected based on potency of inhibiting DPP-IV, their binding affinities with GLP-1R were also analyzed. The peptides posing a strong interaction with GLP-1R were eliminated regardless of their DPP-IV inhibiting potency. The docking protocol was performed by focusing on key active-site residues for receptor binding and activation. These residues are Q234, V237, W306, R310, I313, E387, L388, R190, Y152, T298, R299, N300, D372, R380, L384, L141, L144, Y148, K197, Y205, S392, Y145, W297, Q211, and H212 of GLP-1R. These GLP-1R residues provide interactions with GLP-1 by hydrogen bonds, van der Waals interactions, and hydrophobic interactions. R299, R190, and K197 form hydrogen bonds with GLP-1 residues of H7 and E9; L388 and S392 participate in van der Waals interactions with E9 in GLP-1; T298 and W297 make polar interactions with the neighboring serine pocket of GLP-1 (S14, S17, S18).^15^

Docking protocol on HADDOCK 2.4 was performed to analyze interactions of both shortlisted 26 peptides with DPP-IV and with GLP-1R based on specified active residues. Additionally, commercial peptide-based GLP-1 analogue drugs were tested against DPP-IV and GLP-1R for comparison. Analyzed peptides and drugs with their sources are listed in Table 1. The tool formed possible clusters with queried interactors according to parameters of cluster size, RMSD from the overall lowest-energy structure, van der Waals energy, electrostatic energy, desolvation energy, restraints violation energy, buried surface area, and Z-score. The highest-ranked cluster’s PDB-formatted files for each query were submitted to the PRODIGY (https://wenmr.science.uu.nl/) online tool. Under the protein–protein tab, DPP-IV or GLP-1R was described as interactor 1; candidate peptides were described as interactor 2. The binding temperature was determined as 36.85 °C to mimic the human body, and the PRODIGY run was performed for each peptide. Binding affinity was determined by binding free energy (Δ*G*) in kcal/mol values collected by PRODIGY queries. Thereafter, the Residue Interaction Network Generator (RING) (https://ring.biocomputingup.it/) web tool was used to identify noncovalent interactions at the atomic level in protein structures. PDB files of docking output clusters were loaded into the RING; hydrogen and water were included; other parameters were stayed as default and submitted to identify interactions.

### 2.3 ADMET Profiling and Stability Screening

Previously identified peptide sequences were converted to the Simplified Molecular Input Line Entry System (SMILES) structural format by using the PepSMI (https://www.novoprolabs.com/tools/convert-peptide-to-smiles-string) web tool, which is an algorithm that provides a string of text from raw sequences of the queried molecules.^16^ After that, ADMET profiling of the peptides was performed using the latest version (January 2024 Release) of the ADMETlab 3.0 web tool (https://admetmesh.scbdd.com/). The SMILES files of the candidate peptides were submitted to the platform. Collected scores were compared with the scores of native GLP-1, approved DPP-IV inhibitors, and GLP-1 analogue drugs. Additionally, the stability of peptides was calculated using the Expasy ProtParam (https://web.expasy.org/protparam/) web tool. The full length of peptide sequences was loaded and queried; an instability index was computed based on the full length of the peptide and previously assigned weight value of instability to each of 400 different known dipeptides.^17^

### 2.4 Molecular Dynamics Simulation

Molecular dynamics (MD) simulations of DPP-IV were performed for the apo protein and two DPP-IV-peptide complexes (peptide 3 and peptide 10) using GROMACS (version 2024.1). Protein/peptide topologies were generated with pdb2gmx employing the AMBER99 force field and the TIP3P water model. Each system was placed in a cubic periodic simulation box with a minimum solute–box distance of 0.5 nm, solvated with explicit water, and neutralized with Na^+^/Cl^–^ while additionally adding NaCl to a final concentration of 0.15 M. Following solvation and ion addition, the systems were subjected to a two-stage energy minimization (steepest descent followed by conjugate gradient), equilibrated sequentially under NVT and NPT conditions, and then propagated in a production run of 500 ns. Trajectories were postprocessed to correct for periodic boundary conditions and centered prior to analysis. Structural stability and dynamics were quantified using RMSD, RMSF (per residue), radius of gyration, and solvent-accessible surface area (total and per residue) computed with standard GROMACS analysis tools; intermolecular hydrogen bonding was monitored over time, and secondary-structure evolution was evaluated using DSSP.

### 2.5 *In Vitro* Inhibition Assay

Inhibition activity of candidate peptides against DPP-IV was previously evaluated by *in silico* methods. Among these peptides, peptide 3 was also tested for its *in vitro* activity to test whether it suppresses proteolytic activity of DPP-IV. For this purpose, a Dipeptidyl Peptidase IV (DPP-IV) Inhibitor Screening Assay Kit (E-BC-D007) was purchased from Elabscience Biotechnology Co., Ltd. (Wuhan, China). The manufacturer’s instructions were followed without modifications. Peptide solutions were prepared at the decided concentrations (25, 50, 100, 200 μM). In a 96-well plate, 20 μL of enzyme working solution and 30 μL of peptide solution were added to each well, incubated at 37 °C for 10 min, followed by adding 170 μL of reaction working solution. The well plate was sealed with foil and incubated at 37 °C for 30 min. Fluorescence intensity was measured on a Glomax Multi Elisa Reader (Promega Corporation, Wisconsin, USA) at an excitation wavelength of 360 nm and an emission wavelength of 460 nm. Sitagliptin was used as a positive control since it is a commercial DPP-IV inhibitor provided by the kit. The inhibition rate was calculated based on eq 1 in the manual:

1 $$\mathrm{Inhibition}\;\mathrm{Rate}\;\left(\%\right)=\left(F_{\mathrm{control}}-F_{\mathrm{sample}}\right)÷\left(F_{\mathrm{control}}-F_{\mathrm{blank}}\right)\times 100$$

*F*_blank_: The fluorescence intensity of the blank well.

*F*_sample_: The fluorescence intensity of the sample well.

*F*_control_: The fluorescence intensity of the control well.

Measured absorbance values were used in the calculation, and the inhibition capacity of the peptide was analyzed.

### 2.6 Data Analysis

All *in vitro* experiments were performed independently in triplicate. Findings were presented as mean ± standard deviation (SD). Statistical differences between groups were analyzed using one-way analysis of variance (ANOVA) with the Dunnett’s post hoc test. A *p*-value of <0.0001 was considered statistically significant. Data visualization was performed using GraphPad Prism 11.0 (GraphPad Software, San Diego, CA, USA).

## 3 Results

### 3.1 Peptide Library Construction

To establish the candidate peptide library, a comprehensive computational screening was performed across multiple databases, including Swiss-Prot, NCBI, the Antimicrobial Peptide Database (APD), PeptideAtlas, and UniProtKB, followed by local BLASTp analyses. The screening strategy involved utilizing the full-length 30-amino-acid peptide as well as its fragmented forms (17-aa and 13-aa segments). Because of the short length of these query sequences, the probability of obtaining random background matches in large databases is inherently high. Therefore, to filter out false-positive alignments and ensure high statistical significance, a stringent expected value (E-value) threshold was optimized and set to 0.004. Following this alignment, human-derived peptides, sequences with 100% identity to human GLP-1, and low similarity variants were systematically excluded. Ultimately, by prioritizing venom-derived peptides among the remaining significant hits, we refined the initial large library to a final shortlist of 26 candidate peptides.

Among the species selected for the construction of a peptide library, mouse glycosynthetic peptides, cow, and rat did not exhibit any sequence similarity with the three versions of GLP-1. Mouse plasma, zebrafish, *Streptococcus*, yeast combined nonredundant sequences, *S. aureus* COL, *Drosophila*, yeast (*C. albicans*), *Leptospira interrogans*, and *B. burgdorferi* also demonstrated no sequence similarity with the truncated versions containing the 13 amino acids. However, *M. tuberculosis* and *C. elegans* showed sequence similarity only in the complete GLP-1 version of the peptide, which consists of 30 amino acids. *S. aureus* HG001 and *Halobacterium* did not share similarity with the truncated version containing the 17 amino acids. Additionally, *G. gallus* and *P. falciparum* did not exhibit similarity with either the 17-amino-acid truncated version or the GLP-1 version of the peptide. Finally, honeybee and *Leptospira interrogans* showed no sequence similarity with the nontruncated GLP-1.

### 3.2 Molecular Docking Analysis

Based on HADDOCK 2.4 PRODIGY queries, binding affinities of each peptide toward DPP-IV or GLP-1R are listed in Table 1 along with their sources and sequences. Peptide 3 and peptide 10 possessed the strongest binding affinities to DPP-IV among 26 candidates according to Δ*G* (kcal/mol) values representing the binding free energy. Binding energy between native GLP-1 with DPP-IV and GLP-1R, −12.4 and −11.2 kcal/mol, respectively, was considered as a reference since it is the biological ligand for both proteins in the human body. Binding free energy between peptide 3 and DPP-IV was predicted as −15.6 kcal/mol, and peptide 3 had a stronger interaction with DPP-IV compared with DPP-IV binding with native GLP-1. Similarly, peptide 10 also showed a stronger interaction, −16.5 kcal/mol binding free energy, with DPP-IV compared with native GLP-1. Their binding affinity scores with GLP-1R were not stronger than native GLP-1 affinity to the receptor. The binding energies for peptides 3 and 10 toward GLP-1R were −9.7 and −11.4 kcal/mol, respectively, indicating less binding energy for peptide 3 and slightly higher energy for peptide 10 compared with native GLP-1 (−11.2 kcal/mol). The commercial pharmaceutical peptides also exhibited strong affinities for both DPP-IV and GLP-1R, as expected. Their binding free energy values were similar to peptide 3 and peptide 10. All taken together, two candidate peptides, peptide 3 and peptide 10, have the potency to inhibit DPP-IV with a stronger affinity without occupying GLP-1R.

Binding affinity scores showed peptide 3 and peptide 10 as the most promising peptides in terms of their interaction with DPP-IV. As further analysis, their interacting residues identified by the RING tool are shown in Table 2. DPP-IV was identified as chain A as source; queried peptides were identified as chain B as target. Each source and target residue was described for each interaction type, including hydrogen bond, van der Waals interaction, ionic bond, and π-hydrogen bond. RING tool analysis revealed that the peptides had strong affinities to bind with DPP-IV via various interactions (Table 2). The 3D models of residue-specific interactions are also shown in Figures 1 and 2. Some of the key residues, such as Arg125 and Tyr120, were observed to have hydrogen bonds with DPP-IV for peptide 3. Noncovalent interactions such as an ionic bond between ASP-556 (DPP-IV) and LYS-23 (peptide 3), and a π-hydrogen bond between TRP-629 (DPP-IV) and THR-18 (peptide 3) were also observed for peptide 3. In addition, various amino acid residues had van der Waals interactions with peptide 3. Similarly, some of the key amino acid residues, including Ser101, Asp192, and Lys250, showed hydrogen bonding with DPP-IV for peptide 10. An ionic bond between LYS-250 (DPP-IV) and GLU-36 (peptide 10) was also observed. Additionally, various amino acid residues such as TYR-48, LYS-50, PHE-240, THR-251, SER-744, and SER-745 had van der Waals interactions with peptide 10.

**Figure 1:**
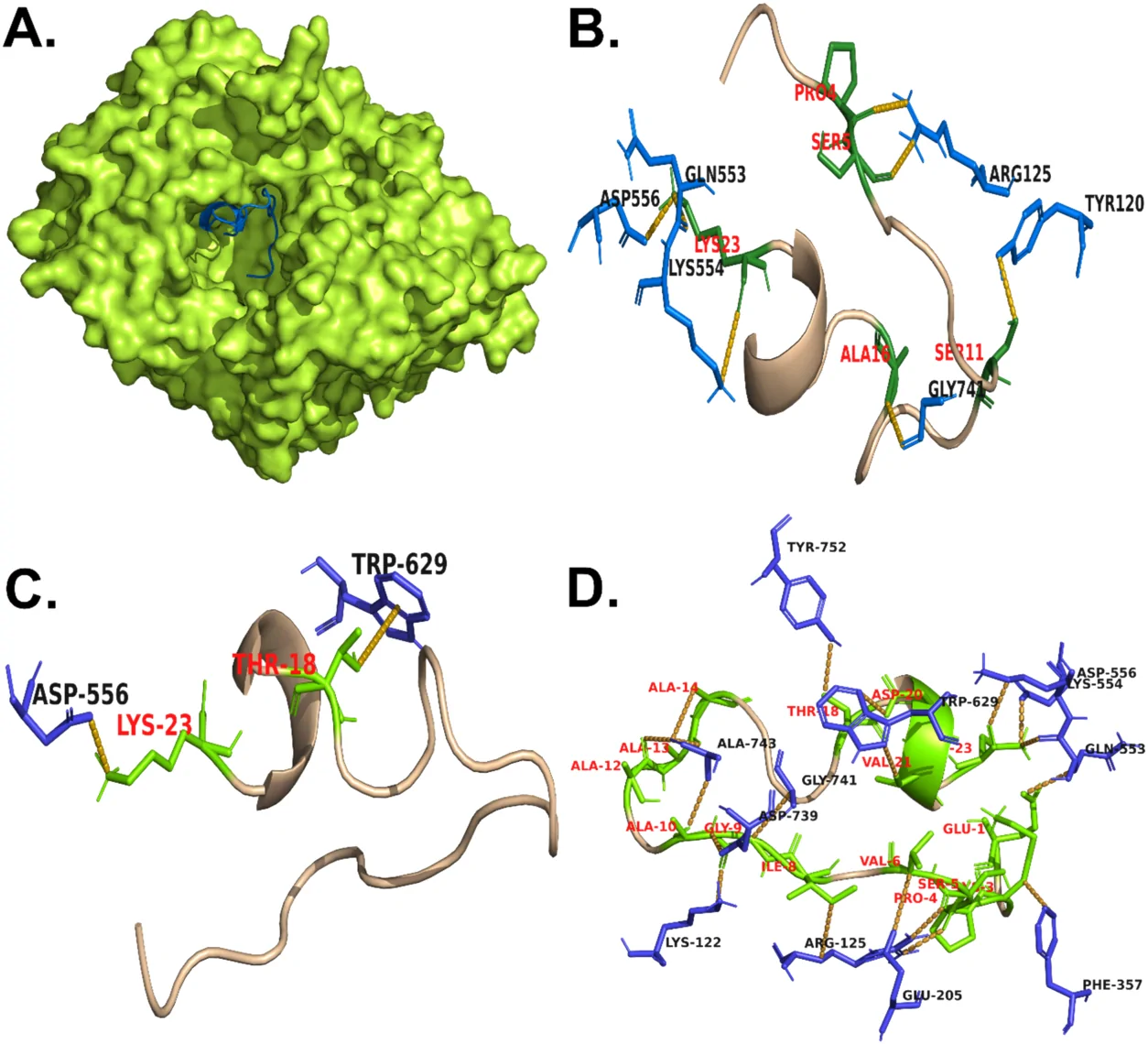
3D docking models of peptide 3 complexed with DPP-IV. (A) Surface model of DPP-IV (green) docked with peptide 3 (blue) within the predicted binding pocket. (B) Hydrogen bond contact between peptide 3 and DPP-IV. Residues of DPP-IV involved in hydrogen bonding are shown in blue; residues of peptide 3 joining hydrogen bonding are highlighted in green. (C) Noncovalent interactions: an ionic bond between ASP-556 (DPP-IV) and LYS-23 (peptide 3), and a π-hydrogen bond between TRP-629 (DPP-IV) and THR-18 (peptide 3). (D) van der Waals contacts between residues of peptide 3 (green) and residues of DPP-IV (blue).

**Figure 2:**
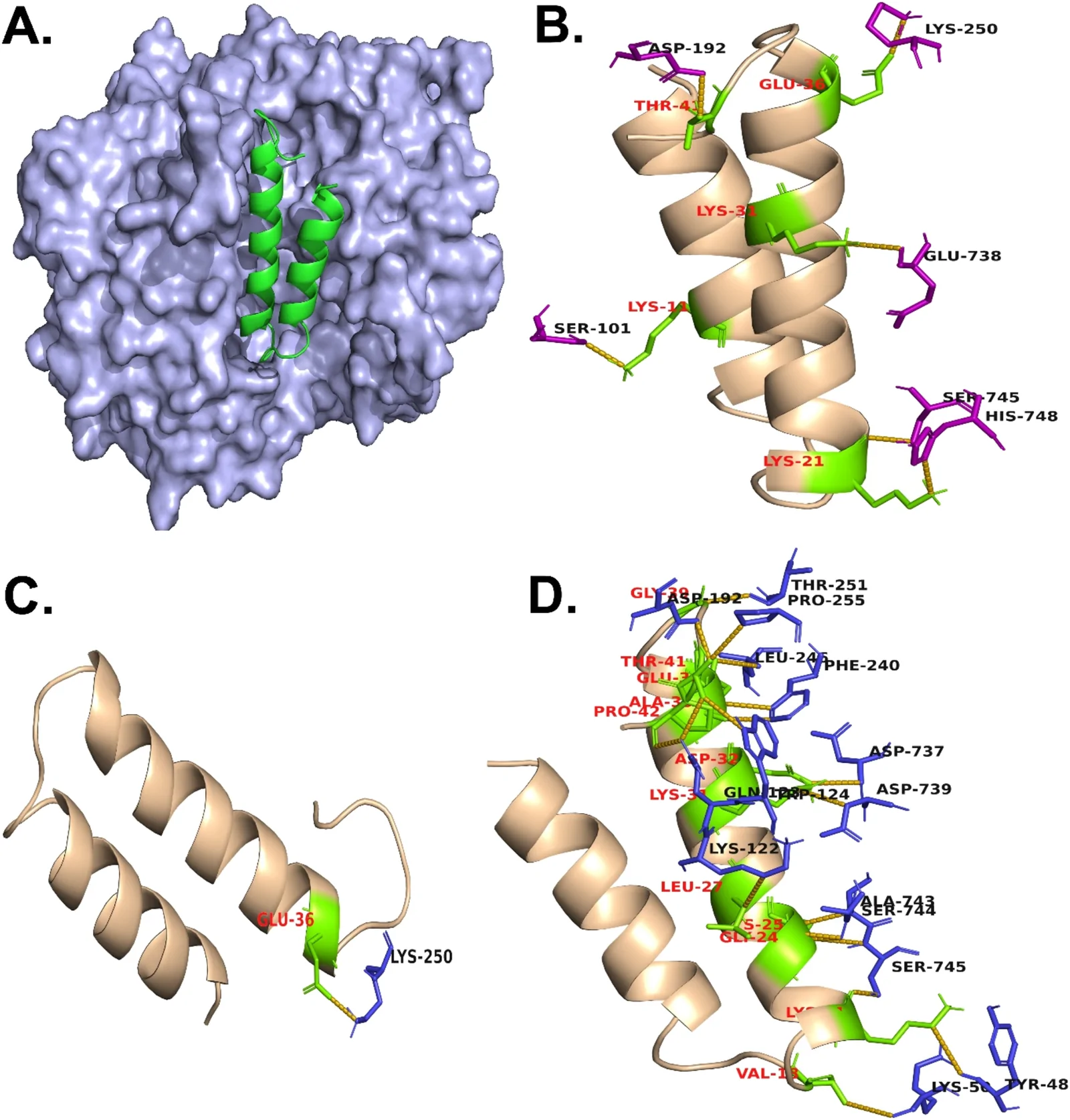
3D docking models of peptide 10 complexed with DPP-IV. (A) Surface model of DPP-IV (blue) docked with peptide 10 (green) within the predicted binding pocket. (B) Hydrogen bond contact between peptide 10 and DPP-IV. Residues of DPP-IV involved in hydrogen bonding are shown in purple; residues of peptide 10 joining hydrogen bonding are highlighted in green. (C) Noncovalent interactions: an ionic bond between LYS-250 (DPP-IV) and GLU-36 (peptide 10) identified using the RING 4.0 tool. (D) van der Waals contacts between residues of peptide 10 (green) and residues of DPP-IV (blue).

**Table 2:** Interacting Residues between Peptide 3 and DPP-IV as well as Peptide 10 and DPP-IV

peptide 3			peptide 10		
source	interaction^a^	target	source	interaction^a^	target
A/120/TYR	HBOND	B/11/SER	A/101/SER	HBOND	B/11/LYS
A/125/ARG	HBOND	B/4/PRO	A/192/ASP	HBOND	B/41/THR
A/125/ARG	HBOND	B/5/SER	A/250/LYS	HBOND	B/36/GLU
A/553/GLN	HBOND	B/23/LYS	A/738/GLU	HBOND	B/31/LYS
A/554/LYS	HBOND	B/23/LYS	A/745/SER	HBOND	B/21/LYS
A/556/ASP	HBOND	B/23/LYS	A/748/HIS	HBOND	B/21/LYS
A/741/GLY	HBOND	B/16/ALA	A/250/LYS	IONIC	B/36/GLU
A/556/ASP	IONIC	B/23/LYS	A/48/TYR	VDW	B/21/LYS
A/629/TRP	PIHBOND	B/18/THR	A/50/LYS	VDW	B/18/VAL
A/122/LYS	VDW	B/9/GLY	A/122/LYS	VDW	B/27/LEU
A/125/ARG	VDW	B/4/PRO	A/123/GLN	VDW	B/41/THR
A/125/ARG	VDW	B/5/SER	A/123/GLN	VDW	B/42/PRO
A/125/ARG	VDW	B/8/ILE	A/124/TRP	VDW	B/41/THR
A/205/GLU	VDW	B/6/VAL	A/192/ASP	VDW	B/41/THR
A/357/PHE	VDW	B/3/LYS	A/240/PHE	VDW	B/35/ALA
A/553/GLN	VDW	B/1/GLU	A/240/PHE	VDW	B/36/GLU
A/553/GLN	VDW	B/23/LYS	A/246/LEU	VDW	B/36/GLU
A/554/LYS	VDW	B/23/LYS	A/251/THR	VDW	B/39/GLY
A/556/ASP	VDW	B/23/LYS	A/255/PRO	VDW	B/41/THR
A/629/TRP	VDW	B/20/ASP	A/737/ASP	VDW	B/32/ASP
A/629/TRP	VDW	B/21/VAL	A/739/ASP	VDW	B/31/LYS
A/739/ASP	VDW	B/10/ALA	A/743/ALA	VDW	B/24/GLY
A/741/GLY	VDW	B/9/GLY	A/743/ALA	VDW	B/25/LYS
A/743/ALA	VDW	B/10/ALA	A/744/SER	VDW	B/25/LYS
A/743/ALA	VDW	B/12/ALA	A/745/SER	VDW	B/21/LYS
A/743/ALA	VDW	B/13/ALA	A/745/SER	VDW	B/25/LYS
A/743/ALA	VDW	B/14/ALA	-	-	-
A/752/TYR	VDW	B/18/THR	-	-	-

a VDW: van der Waals interaction; HBOND: hydrogen bond; IONIC: ionic bond; PIHBOND: π-hydrogen bond.

### 3.3 ADMET and Stability Screening

Since the primary parameter of DPP-IV inhibitor determination is binding free energy values, peptides 3 and 10 were selected as the most promising ones for further evaluations. Thus, pharmacokinetic characteristics of those peptides were assessed by ADMET 3.0, including various parameters such as Caco-2 permeability, plasma protein binding (PPB), CYP1A2 inhibition, plasma clearance, half-life duration, and toxicity, together with protein stability, which was computed by Expasy ProtParam (Table 3). Their profiles are compared with native GLP-1, dulaglutide (commercial GLP-1 analogue drug approved by FDA), and sitagliptin (DPP-IV inhibitor approved by FDA).

**Table 3:** Pharmacokinetic Properties of Selected Peptides

category	property (unit)	dulaglutide	native GLP-1	peptide 3	peptide 10	sitagliptin	inference/references range
Absorption	Caco-2 permeability (cm/s)	–6.78	–6.938	–7.882	–10.53	–5.148	Optimal: higher than −5.15
Distribution	Plasma protein binding (%)	–8.3	–8.7	–7.6	–41.8	58.0	Optimal: <90%
Metabolism	CYP1A2 inhibitor	0.000	0.000	0.000	0.000	0.000	>0.5: An inhibitor
							<0.5: Non-inhibitor
Excretion	CL_plasma_ (mL/min/kg)	0.429	0.31	0.823	–0.197	6.519	>15 mL/min/kg: high clearance
							5–15 mL/min/kg: moderate clearance
							<5 mL/min/kg: low clearance
	*T*_1/2_ (hours)	1.785	1.962^a^	1.65	4.334	0.715	Long half-life: > 3 h
							Short half-life: < 3 h
Toxicity	hERG blockers	0.0	0.0	0.0	0.0	0.319	>0.5: Blocker
							<0.5: Non-blocker
	Drug-induced liver injury (DILI)	0.002	0.001	0.0	0.0	0.621	>0.5: Toxic to liver
							<0.5: Nontoxic to liver
	Carcinogenicity	0.0	0.0	0.0	0.0	0.143	>0.5: Carcinogen
							<0.5: Non-carcinogen
	Skin sensitization	1.0	1.0	1.0	1.0	0.988	>0.5: Sensitizer
							<0.5: Non-sensitizer
Protein stability	Instability index	33.12	17.69	8.27	–2.41	-	<40: Stable
							>40: Unstable

a *T*_1/2_ value is 1.962 according to ADMET 3.0, but it is known that rapid degradation by DPP-IV reduces this to 0.03 and is accepted as such in literature studies.^6^

Peptide 3 and peptide 10 showed slightly lower permeability compared with both native GLP-1 and dulaglutide. Sitagliptin, which is an oral medication, had ultimate absorption due to its smaller molecule nature compared with peptides. The lower permeability of peptides might lead to a challenging oral administration, but it can be enhanced by alternative formulation strategies including encapsulation or peptide additive molecules.^18,19^ Although the distribution attitudes of both peptide 3 and 10 were favorable for bioavailability, peptide 3 exhibited a better distribution profile as it showed a low plasma protein-binding percentage within the suggested range. In ADMET analysis, it is accepted that a binding percentage lower than 90% is optimal since drugs with high protein binding may have a low therapeutic index. Higher binding rates, as seen in sitagliptin, can affect the free drug concentration and efficacy. None of the peptides inhibited the CYP1A2 enzyme, which is promising to avoid drug–drug interactions, and the metabolic stability of the compound is ensured. Apart from sitagliptin, all peptides’ plasma clearance values were in the optimal range and, together with their prolonged half-life, showed that their excretion profile is promising. Also, both peptide 3 and peptide 10 exhibited no hERG blockage, no drug-induced liver injury (DILI), and no carcinogenicity, indicating that they are safe and not cardiotoxic compounds. Additionally, the stabilities of the peptides were assessed and the output values were below 40 for both peptides, indicating that both are stable peptides.^20^

### 3.4 Molecular Dynamics Simulation

The dynamic stability and structural integrity of the DPP-IV enzyme in complex with peptides 3 and 10 were evaluated by performing 500 ns MD simulations. In order to understand the inhibitory strength of the peptides, the results were compared against the apo form of DPP-IV.

#### 3.4.1 System Stability, Conformational Change, Flexibility, and Fluctuations Evaluation

The 500 ns MD simulation reveals distinct dynamic profiles for the two candidate peptides. First, root mean square deviation (RMSD) was used to evaluate the structural stability of the complexes, which reached an equilibrium state within the first 50 ns of the simulation (Figure 3). The free protein was stabilized at an RMSD value of approximately 0.25 nm. It maintained a highly stable, steady plateau between 50 and 450 ns with a slight fluctuation around 0.26–0.29 nm at the end of the simulation. Similarly, peptide 10 demonstrated structural stability, tightly mirroring the trajectory of the free protein with an average RMSD of ∼0.28 nm. On the other hand, peptide 3 complexed with DPP-IV exhibited structural flexibility and instability with a significantly higher RMSD baseline (∼0.40–0.45 nm) and a major conformational transition at 330 ns compared with the free protein. This suggests that peptide 3 can induce substantial conformational stress or pocket distortion in the target protein.

**Figure 3:**
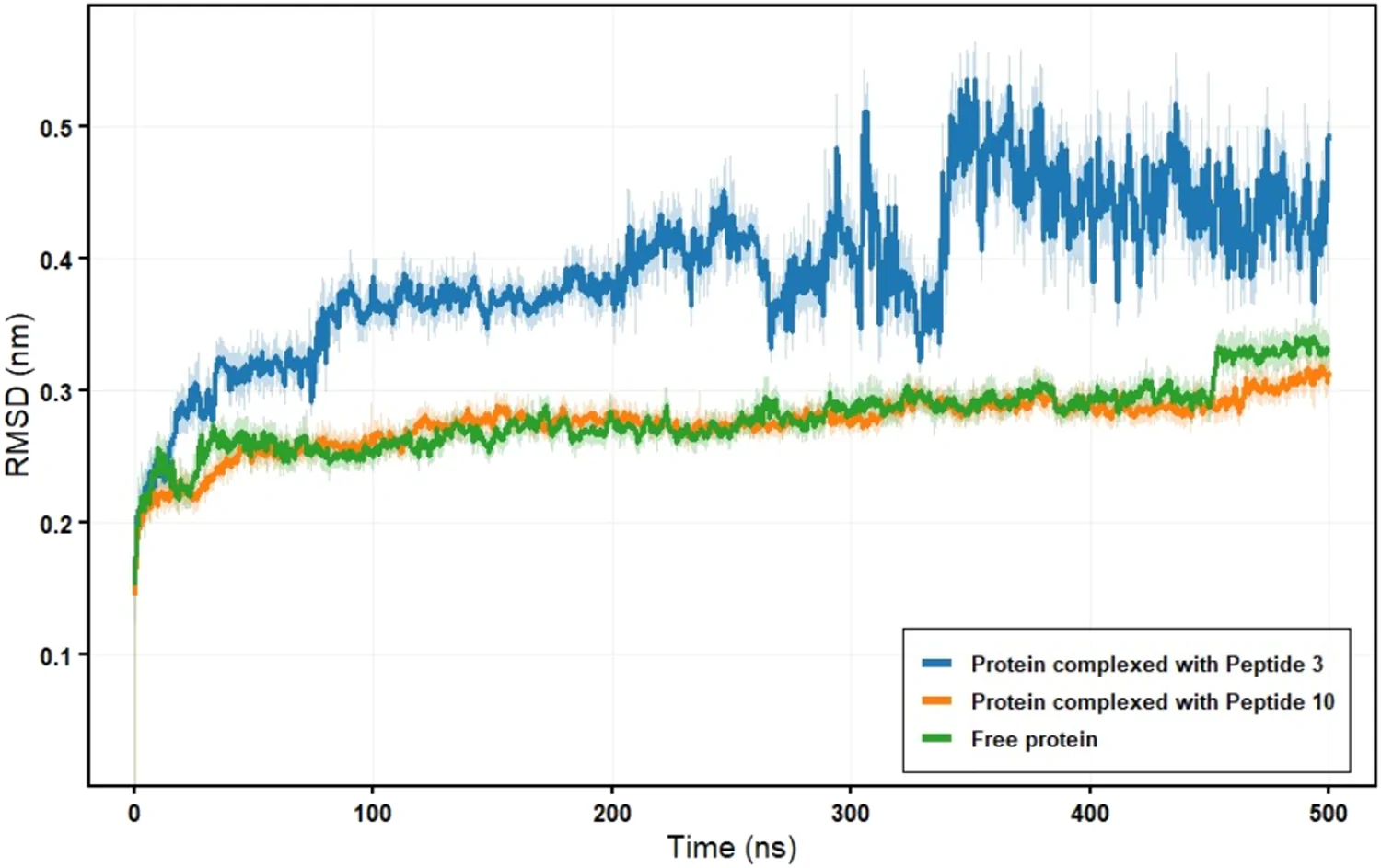
Molecular dynamics simulation RMSD analysis over a 500 ns simulation. Time-dependent RMSD of the backbone for free DPP-IV and peptide-bound DPP-IV complexes was calculated over the simulation time to evaluate structural stability and conformational changes upon peptide binding.

To identify the specific residue-level flexibilities driving the overall structural variations, the root mean square fluctuation (RMSF) of the protein backbone was analyzed across the 500 ns trajectory (Figure 4). The core regions of the protein remained highly stable across all systems, maintaining a baseline fluctuation below 0.2 nm. However, significant local flexibility was observed in the region spanning around 30 residues between 230 and 260 (Figure 4). In peptide 10 complexed with DPP-IV, fluctuations within this region were closely similar to those of the free protein. This indicated that binding does not disrupt native loop dynamics. In contrast, binding of peptide 3 induced dramatic local hyper-flexibility, with fluctuations spiking drastically to ∼1.4 nm at residue 245, accompanied by a secondary peak at residues 270–290 (∼0.6 nm). This localized structural destabilization can be correlated directly with the elevated global RMSD and indicates that peptide 3 might promote a substantial conformational rearrangement or pocket distortion within these specific residue clusters.

**Figure 4:**
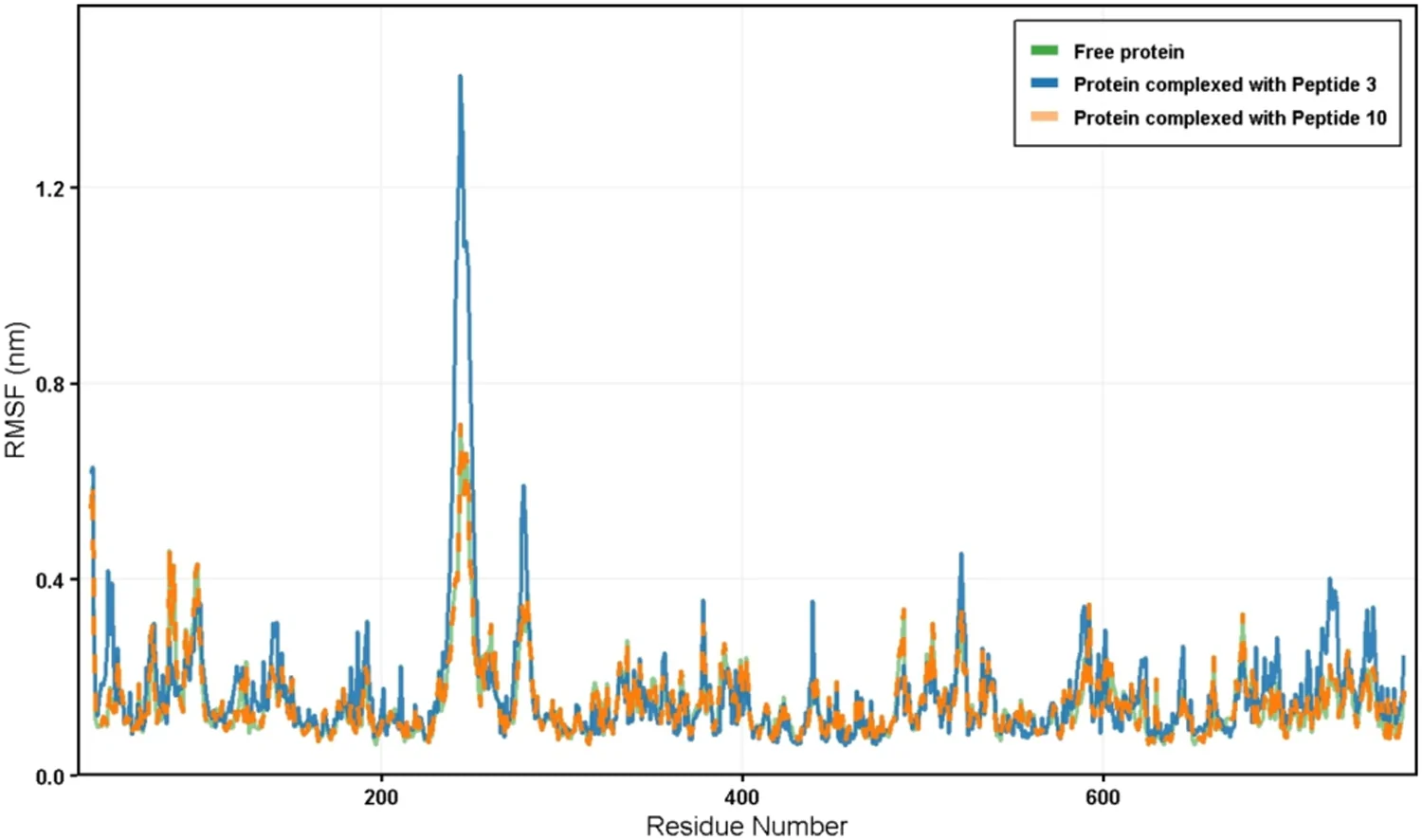
Root mean square fluctuation (RMSF) of DPP-IV residues in the apo-state and bound-state. The analysis was performed to evaluate residue-level flexibility and to identify regions affected by peptide binding.

Additionally, the radius of gyration (*R*_g_) was calculated over the 500 ns simulation to assess the effect of peptide binding on the overall compactness and structural integrity of DPP-IV (Figure 5). The free protein maintained a highly compact native conformation with a baseline *R*_g_ of ∼2.68 nm. Binding of peptide 10 resulted in a minor, uniform baseline shift (∼2.71 nm) but remained remarkably stable and consistent throughout the simulation. However, the peptide 3 complex exhibited pronounced structural expansion and instability. This expansion indicates that the hyper-flexibility observed at residues 230–260 may induce a temporary large-scale domain opening or structural relaxation. Solvent-accessible surface area (SASA) was also monitored for further investigation on the structural variations (Figure 6). The free protein exhibited the lowest solvent exposure, maintaining a steady baseline between 310 and 325 nm^2^. In the peptide 10 complex, SASA shifted slightly higher to a highly stable plateau of ∼325–330 nm^2^, indicating a well-preserved structural shield with minimal conformational disruption. Conversely, the peptide 3 complex demonstrated a distinct, time-dependent increase in solvent exposure. SASA of the peptide 3 complex steadily climbed after 260 ns, peaking at ∼340–344 nm^2^. This inflation in SASA also validates that the hyper-flexibility at residues 230–260 can induce a significant domain-opening event, expanding the protein’s boundary and exposing a larger surface area to the surrounding solvent environment before temporarily restabilizing after 440 ns. Overall, these findings demonstrated that peptide 10 formed a structurally sound, highly compact, and stable complex capable of preserving the target protein’s native framework, while peptide 3 induced severe conformational stress, leading to localized hyper-flexibility at residues 230–260 and an unfavorable domain opening.

**Figure 5:**
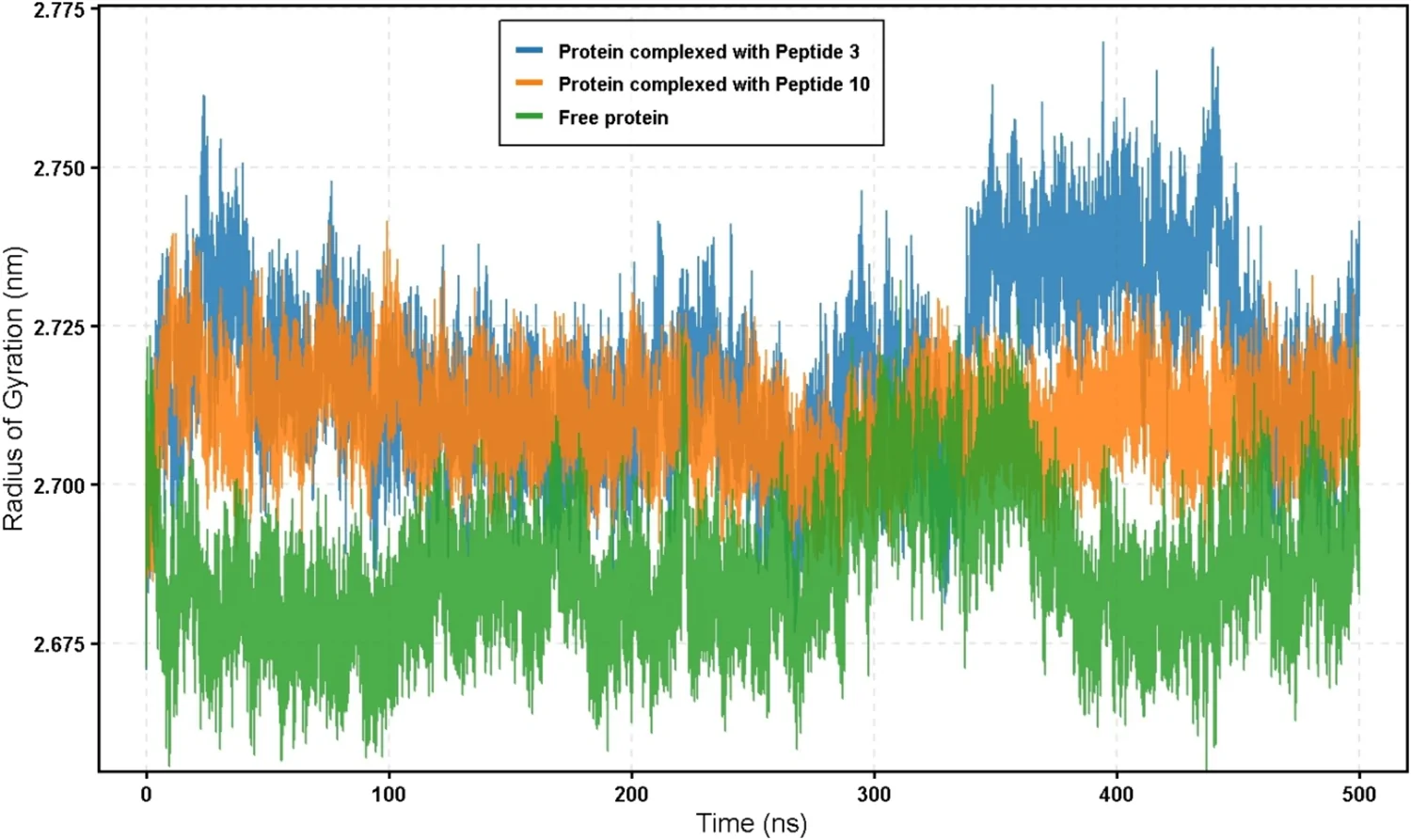
Radius of gyration (*R*_g_) measurement of free protein and peptide-bound complexes throughout the 500 ns trajectory. *R*_g_ values were calculated over the simulation time to assess the overall compactness and structural stability of the protein upon peptide binding.

**Figure 6:**
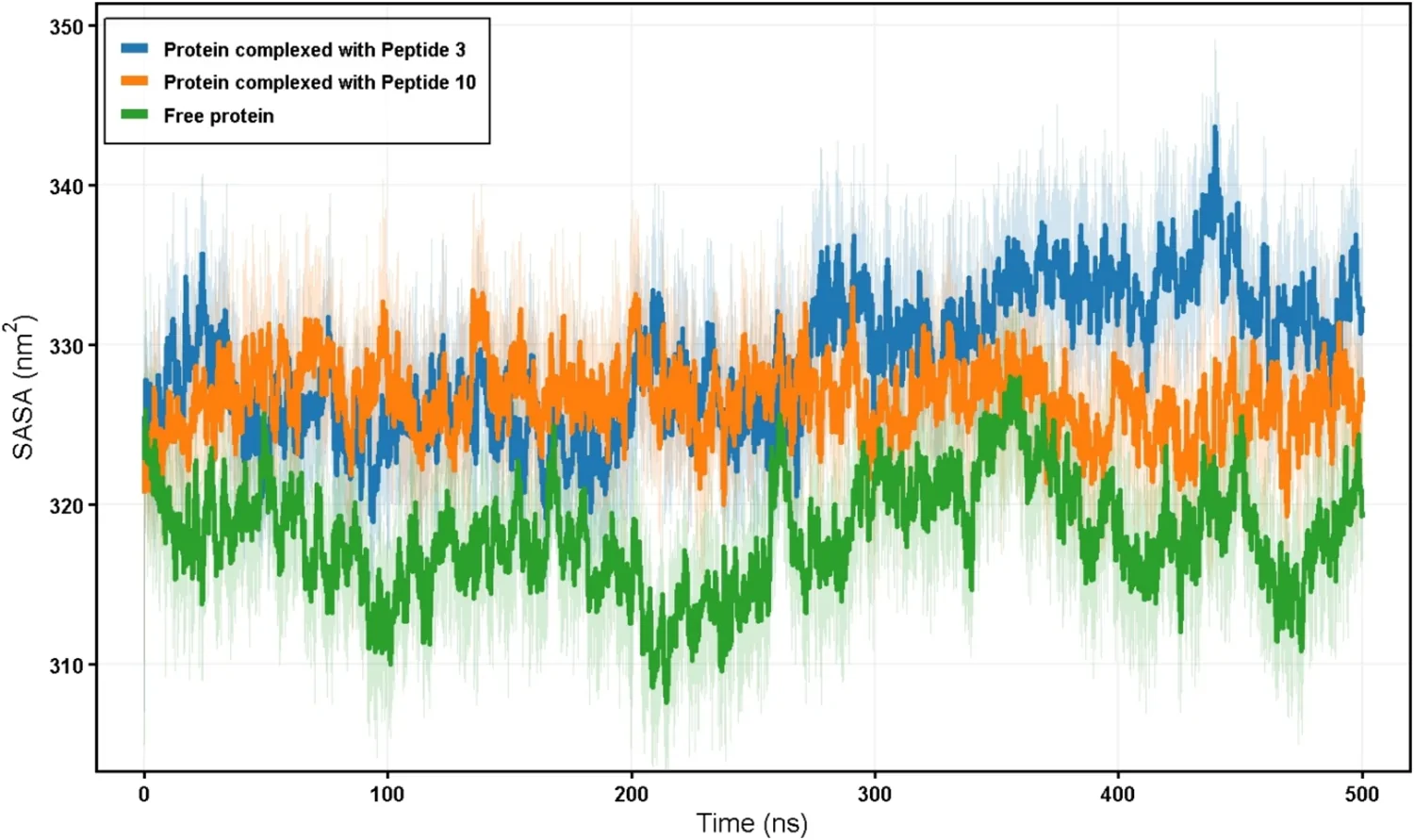
Total surface area of DPP-IV accessible to the solvent over 500 ns, reflecting the degree of active-site shielding by the inhibitors. SASA analysis of free DPP-IV and peptide-bound complexes reflects changes in the exposure of the protein surface and provides insight into conformational rearrangements induced by peptide binding.

#### 3.4.2 Intermolecular Interaction Analysis

The specific intermolecular forces driving the binding affinity and structural stability of the complexes, including hydrogen bonding (Figure 7A) and van der Waals (vdW) contacts (Figure 7B) between the peptides and DPP-IV, were monitored across the 500 ns trajectory. Peptide 10 established a robust and highly dense interaction network and maintained an average of 12–15 H-bonds that optimized further to 18–20 H-bonds after 380 ns, paired with a consistent baseline of 15–22 vdW contacts. This denoted geometric complementarity and strong polar anchoring. Conversely, peptide 3 displayed a drastically constrained interaction footprint, averaging fewer H-bonds (∼9) and nominal hydrophobic packing (<10 vdW contacts). At around 350 ns, the number of vdW contacts for peptide 3 had a sharp decline. This sudden reduction in steric interaction perfectly coordinates with the previously observed spikes in global RMSD, localized loop fluctuations (residues 230–260), and radius of gyration expansion.

**Figure 7:**
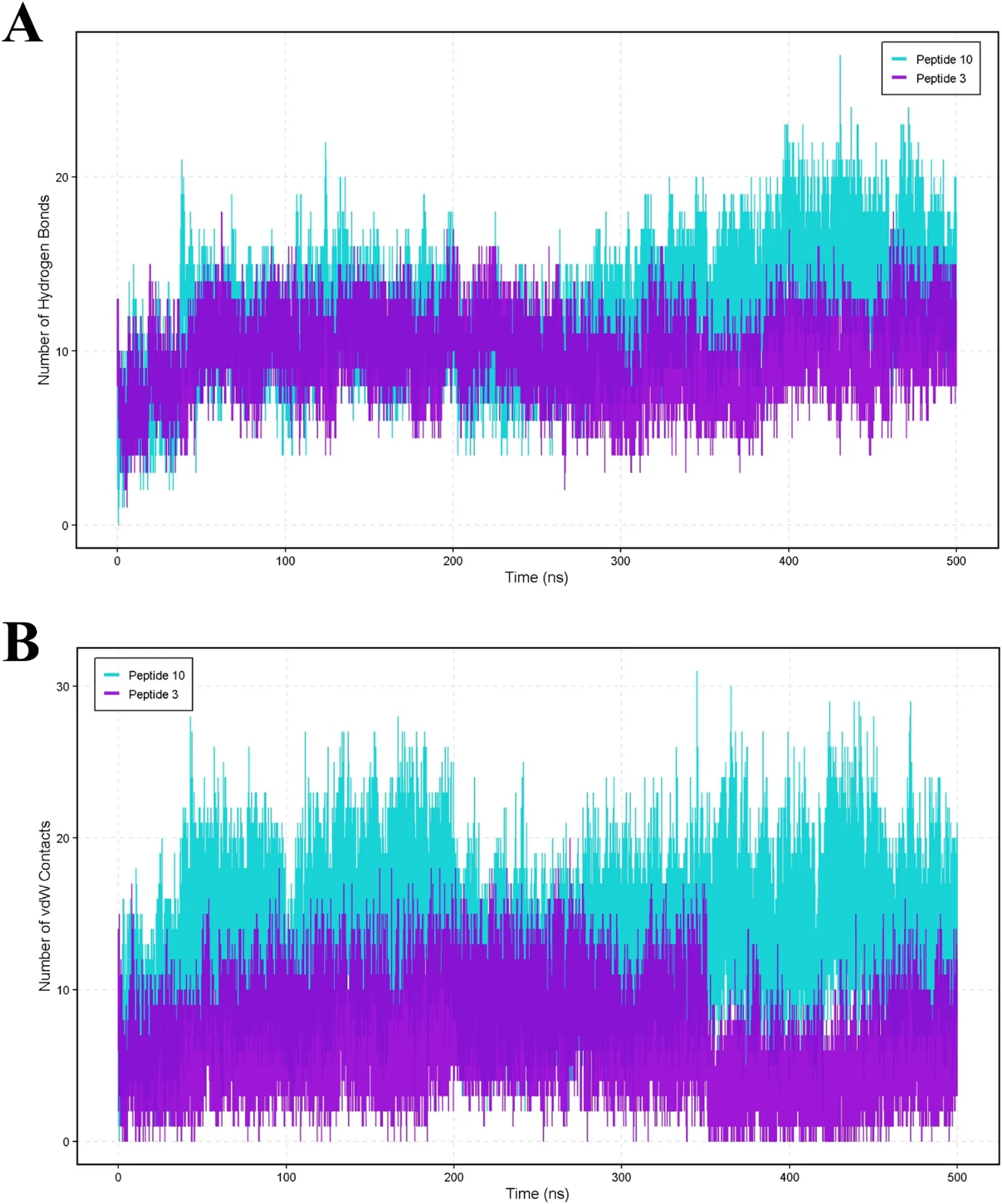
Intermolecular interaction analysis over 500 ns. (A) Hydrogen bond contact analysis of DPP-IV-peptide 3 and DPP-IV-peptide 10 complexes. (B) van der Waals interaction analysis of DPP-IV-peptide 3 and DPP-IV-peptide 10 complexes.

### 3.5 *In Vitro* Inhibition Assay

In addition to the *in silico* analyses, peptide 3 was selected for preliminary *in vitro* evaluation based on a multiparameter prioritization strategy. Although peptide 10 showed a slightly more favorable predicted binding free energy toward DPP-IV, peptide 3 also showed strong predicted DPP-IV binding and displayed a larger predicted difference between its binding affinities toward DPP-IV and GLP-1R. Peptide 3 also showed a relatively less unfavorable predicted Caco-2 permeability value than that of peptide 10. Therefore, peptide 3 was selected as the initial candidate for experimental proof-of-concept validation, while peptide 10 was retained as a high-priority candidate for a subsequent *in vitro* investigation.

The inhibitory strength of peptide 3 against DPP-IV and dose–response profile were determined by testing at various concentrations (25–200 μM). Sitagliptin was used as a positive control to validate the assay conditions. As shown in Figure 8, peptide 3 exhibited a limited inhibitory activity in a dose-dependent manner. The highest concentration, 200 μM, achieved an inhibition of 36.2%; reducing concentrations to 100, 50, and 25 μM resulted in decreasing inhibitory effects, showing values of 31.5, 21.05, and 16.62%, respectively, according to eq 1. The reference commercial inhibitor, sitagliptin, showed almost total suppression of DPP-IV enzymatic activity, reaching 98.74% inhibition under the same experimental conditions. Peptide 3 did not demonstrate inhibitory potency comparable to that of the commercial drug; the consistent decrease in inhibition with reducing concentrations suggests a specific and concentration-dependent interaction with the target protein. The limited efficacy can be correlated with our MD simulation findings, which demonstrated that peptide 3 induces severe conformational stress and structural expansion upon binding. The observed spike in residue flexibility at positions 230–260 and the subsequent loss of receptor compactness can provide a clear structural rationale for the poor *in vitro* activity.

**Figure 8:**
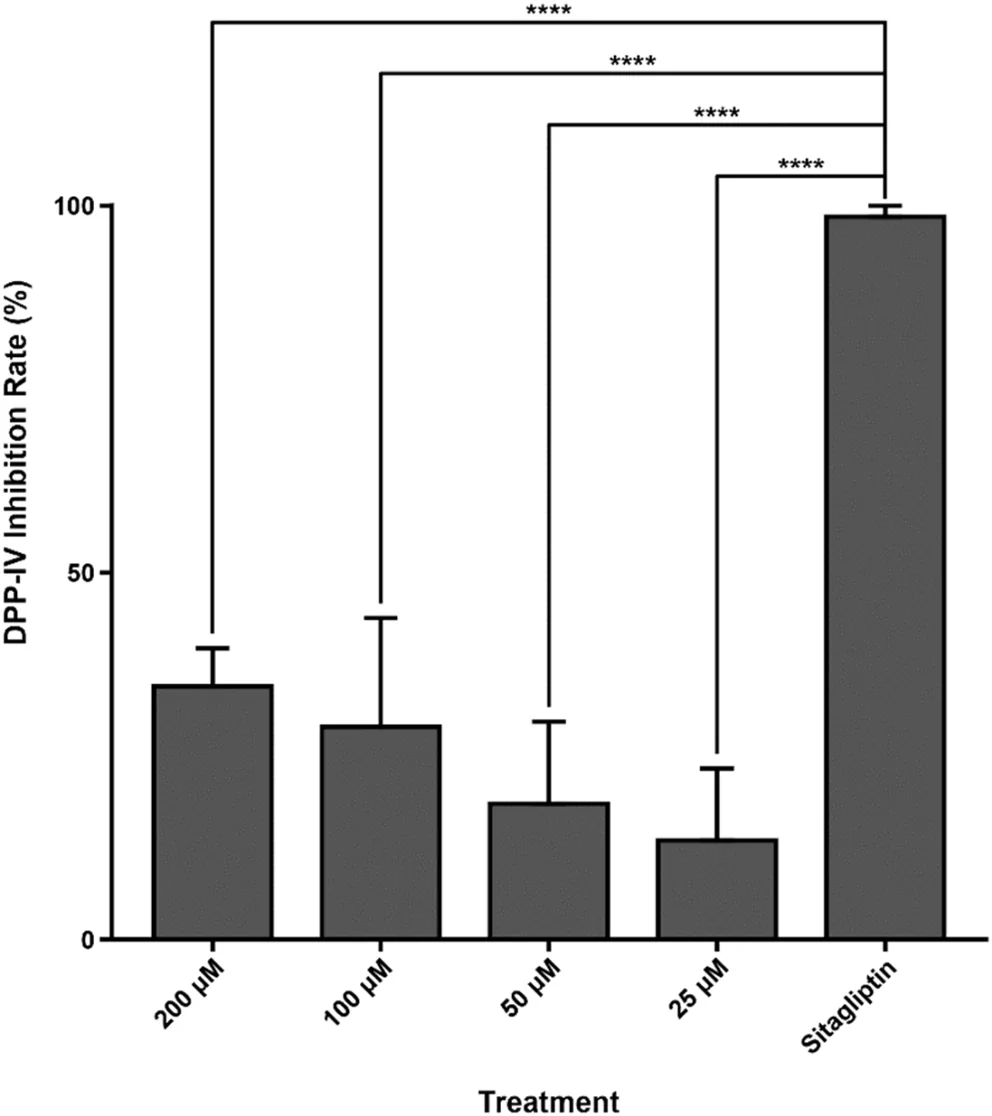
*In vitro* inhibition evaluation of peptide 3 against DPP-IV. Data are represented as mean ± SD of triplicate experiments. Sitagliptin at 100 μM was used as a positive control. One-way ANOVA was used with the Dunnett’s post hoc test for analysis (**** *p* ≤ 0.0001).

## 4 Discussion

The increasing prevalence of T2DM and the high risk of related metabolic diseases have encouraged studies in the field of antidiabetic drugs. GLP-1 analogues are some of the most intensely studied drugs due to their advantages over traditional exogenous insulin injection.^21^ They have a longer half-life due to the amino acid modifications and their increased resistance to DPP-IV cleavage compared with native GLP-1. Additionally, enhanced receptor binding affinity is another significant advantage of the analogues.^22^ Therefore, the investigation of natural peptides showing similarity to native GLP-1 can reveal new antidiabetic compound candidates. Since the degradation of native GLP-1 and its analogues by DPP-IV is a major problem, the binding of peptides to DPP-IV with a strong affinity can prevent protease activity. In the study, 26 shortlisted peptides from various sources were analyzed via molecular docking to discover novel peptide-based DPP-IV inhibitors. The docking analysis revealed that peptides 3 and 10 showed the strongest binding affinities to DPP-IV, with binding free energies of −15.6 and −16.5 kcal/mol, respectively. These two peptides surpassed the native GLP-1, which exhibited a binding energy of −12.4 kcal/mol, in terms of DPP-IV interaction strength, highlighting their selective DPP-IV inhibitory potency. These binding energy scores of both peptides were found to be stronger than those in similar studies with different peptides. For instance, a short peptide (7 amino acids) derived from green seaweed *Ulva* spp. hydrolysates exhibited a binding energy of −7.65 kcal/mol in docking with DPP-IV.^23^ Another study analyzed three antidiabetic peptides from *Cannabis sativa*, and the binding free energy of maximum −11.7 kcal/mol was calculated by PRODIGY.^24^ On the other hand, peptide 3 and peptide 10 exhibited moderate interaction affinity to GLP-1R with −9.7 and −11.4 kcal/mol binding free energies, respectively. The native GLP-1 was found to bind to GLP-1R with a binding energy score of −11.2 kcal/mol. The selective binding profile indicated that both peptides were able to inhibit DPP-IV without interfering with GLP-1R activation. Since the peptides have a dual functionality by inhibiting DPP-IV while maintaining moderate GLP-1R interaction, they could improve GLP-1 half-life by preventing degradation. Ultimately, enhancing the GLP-1 signaling pathway leads to insulin production.

The ADMET profiling demonstrated favorable pharmacokinetic characteristics of peptides, such as low plasma protein binding, since drugs with high protein binding may have a low therapeutic index. Minimal CYP1A2 inhibition and no significant hERG blockage indicated a low toxicity risk. Compared with the native GLP-1 ADMET profile, the candidate peptides showed moderate or better values. Particularly, the half-lives of peptides are much longer than 2 min of native GLP-1, which is known as the major problem in the human body. As supportive findings, previous studies assessing natural peptides, such as hydrolysates of chickpea provicilin and legumin, against DPP-IV showed that the peptides had similar interacting targets to peptides 3 and 10. Also, the pharmacokinetic profile of those peptides exhibited promising circulation, absorption, toxicity, and bioavailability values in addition to gastrointestinal digestion stability. In contrast to peptide 3 and peptide 10, the half-life of the identified peptides was less than 1 h.^25^

MD simulation analysis and experimental evaluation suggested that both peptide 3 and peptide 10 showed DPP-IV inhibitory potency. The 500 ns simulations successfully elucidated the structural stability, core compactness, and intermolecular binding mechanics of DPP-IV complexed with the two candidate peptides, revealing highly distinct conformational profiles. Peptide 10 demonstrated an exceptionally stable and rigid binding mode, tightly maintaining a native-like structural framework characterized by a minimal global deviation, optimal architecture, and steady solvent shielding. This steady-state accommodation was driven by a robust and progressively optimized intermolecular network, peaking at 18–20 hydrogen bonds and over 15 van der Waals contacts. Conversely, peptide 3 exhibited a dynamic, adaptive profile causing induced pronounced localized transitions. Due to its structural brevity and weaker hydrophobic packing, peptide 3 harbors a weaker intermolecular contact network. The transient disruption triggered a sharp global deviation and, driven by massive localized hyper-flexibility at residues 230–260 (the RMSF peak at residue 245), forced a temporary domain-opening expansion of the protein matrix. The RMSF peak at residues 230–260 was previously characterized as the β-propeller “flap” loop and refers to open/close conformation, which is required for normal catalytic activity.^26^ Peptide 3′s binding characteristics are more likely flexible, and its dynamic signature is consistent with substrate-like engagement.^27^ Nevertheless, it is not an irreversible unfolding; there is a recompaction observed after 440 ns in *R*_g_ and SASA analysis shown in Figures 5 and 6. All taken together, peptide 3 can be reported as a reversible gating element displaying higher flexibility instead of a rigid structural element.

The *in vitro* inhibition assay confirmed that peptide 3 possessed a dose-dependent inhibitory effect against DPP-IV, demonstrating limited activity relative to the reference drug, sitagliptin. The inhibition magnitude of peptide 3 is consistent with previous studies covering natural peptides’ DPP-IV inhibitory potency. For example, food-derived natural peptides were tested against DPP-IV and showed 27.7–35.0% inhibition rate at 100–300 μM even with shorter peptide length.^28^ Another study reported modest inhibitory activities for natural peptide-based DPP-IV inhibitors compared with sitagliptin. The peptides derived from *Tenebrio molitor* protein hydrolysates supported that nonoptimized natural peptides require sequence optimization for a similar inhibitory potency to small-molecule inhibitors.^29^ While the experimental data validates its active-site occupancy, the observed global fluctuations in RMSD and the lower density of van der Waals contacts indicate that its truncated structure requires further optimization to match the high-affinity interactions characteristic of sitagliptin. Nevertheless, the comparative molecular dynamics data suggest that introducing greater hydrophobicity, bulky residues, or strategic sequence modifications could significantly maximize biological activity by establishing more stable interactions and denser van der Waals packing. Although downstream *in vitro* evaluation was restricted to peptide 3 due to synthetic and budgetary constraints, the collective findings, including molecular docking scores, favorable pharmacokinetic profiles, long-duration MD simulations, and dose-dependent enzymatic inhibition, support that both peptides could be promising candidates for the development of novel therapeutics targeting the DPP-IV protease.

The experimental evaluation was limited to peptide 3 and, therefore, does not permit a direct comparison of the inhibitory potencies of peptide 3 and peptide 10. Peptide 10 remains an important candidate for future experimental validation because it showed the strongest predicted DPP-IV binding affinity and formed a stable complex during the molecular dynamics simulation. Direct comparative enzyme inhibition assays, including concentration–response experiments and IC_50_ determination for both peptides, will be required to validate their relative inhibitory activities and predicted target preferences. Both candidate peptides will also be tested for their antidiabetic activity by *in vivo* experiments, including diabetic models.

## 5 Conclusion

In this study, we discovered two novel peptide-based DPP-IV inhibitors with the potential to enhance the therapeutic efficacy of GLP-1 in the management of type 2 diabetes mellitus. Novel peptide-based inhibitors of DPP-IV were described in this study with the purpose of enhancing the therapeutic application of GLP-1 in T2DM management. *In silico* screening demonstrated that peptide 3 and peptide 10 exhibited the strongest binding free energies to DPP-IV while making weaker interactions with GLP-1R. ADMET profiling of the peptides confirmed optimal pharmacokinetic properties together with potential stability and bioavailability in general. Molecular dynamics simulations revealed that peptide 10 is a highly stable and structurally optimized lead candidate against DPP-IV, while the dynamic profile of peptide 3 provides vital structural insights into the conformational tolerance and pocket flexibility of the receptor. Peptide 3 was prioritized for subsequent experimental validation because of its better permeability profile and more favorable predicted DPP-IV/GLP-1R binding preference. Peptide 3 demonstrated a 36.2% inhibition of DPP-IV activity *in vitro*. All of these *in silico* and *in vitro* findings support that both peptide 3 and peptide 10 are promising candidates to develop novel DPP-IV inhibitors. To our knowledge, no previous studies have investigated the DPP-IV inhibitory potential of peptide 3 and peptide 10, highlighting their potential as naturally derived antidiabetic peptides. Further *in vitro* and *in vivo* studies are required to validate their DPP-IV inhibitory activity.

## Supplementary Material



## Notes

BLASTp results for the peptides obtained from Swiss-Prot database (Table S1); BLASTp results for the peptides obtained from NCBI database (Table S2); BLASTp results for the peptides obtained from Swiss-Prot database using the 17-amino-acid fragment (Table S3); All hit peptides similar to the full-length GLP-1 sequence (Table S4); All hit peptides similar to the 13-amino-acid fragment of GLP-1 sequence (Table S5); All hit peptides similar to the 17-amino-acid fragment of GLP-1 sequence (Table S6) (XLSX)
